# Identification of the novel role of butyrate as AhR ligand in human intestinal epithelial cells

**DOI:** 10.1038/s41598-018-37019-2

**Published:** 2019-01-24

**Authors:** Ludovica Marinelli, Camille Martin-Gallausiaux, Jean-Marie Bourhis, Fabienne Béguet-Crespel, Hervé M. Blottière, Nicolas Lapaque

**Affiliations:** 1 Micalis Institute, INRA, AgroParisTech, Université Paris-Saclay, 78350 Jouy-en-Josas, France; 2 Sorbonne Universités, UPMC Univ Paris 06, IFD, 4 place Jussieu, 75252 Paris, cedex 05 France; 3 University Grenoble Alpes, CNRS, CEA, IBS, F-38000 Grenoble, France; 4 MetaGenoPolis, INRA, Université Paris-Saclay, 78350 Jouy en Josas, France

## Abstract

The ligand activated transcription factor, aryl hydrocarbon receptor (AhR) emerged as a critical regulator of immune and metabolic processes in the gastrointestinal tract. In the gut, a main source of AhR ligands derives from commensal bacteria. However, many of the reported microbiota-derived ligands have been restricted to indolyl metabolites. Here, by screening commensal bacteria supernatants on an AhR reporter system expressed in human intestinal epithelial cell line (IEC), we found that the short chain fatty acid (SCFA) butyrate induced AhR activity and the transcription of AhR-dependent genes in IECs. We showed that AhR ligand antagonists reduced the effects of butyrate on IEC suggesting that butyrate could act as a ligand of AhR, which was supported by the nuclear translocation of AhR induced by butyrate and *in silico* structural modelling. In conclusion, our findings suggest that (i) butyrate activates AhR pathway and AhR-dependent genes in human intestinal epithelial cell-lines (ii) butyrate is a potential ligand for AhR which is an original mechanism of gene regulation by SCFA.

## Introduction

The mammalian gastrointestinal (GI) tract is colonised by a complex microbial community, referred as gut microbiota. It is well established that host-commensal bacteria crosstalk provides numerous functions for the overall host wellbeing, through the production of microbial metabolites. The host-microbiota interaction is particularly substantial for mucosal barrier functions as well as the development and maintenance of the mucosal immune system^1^. Metabolites derived from commensal bacteria described to strongly impact mucosal homeostasis include the short-chain fatty acids (SCFA), originated from the fermentation of dietary fibres, and indoles, from the degradation of dietary tryptophan. The targeted host receptors of these bacterial products include the cell-surface G-protein-coupled receptors GPR41, GPR43, and GPR109A and nuclear receptors, such as the aryl hydrocarbon receptor (AhR), the pregnane X receptor (PXR), and the farnesoid X receptor (FXR). The receptor-metabolite interactions induce signalling pathways that modulate host gene expression and collectively impact on host metabolism and immune responses^1^.

Recently AhR, a ligand activated transcription factor, has gained considerable attention as a crucial modulator of mucosal immune and metabolic processes, especially in the context of diet and microbiota crosstalk with the host^2–4^. AhR is a member of the basic helix-loop-helix PER-ARNT-SIM (bHLH-PAS) family, initially identified as a hepatic intracellular protein that bounds with high affinity the environmental halogenated contaminant 2,3,7,8-tetrachlorodibenzo-p-dioxin (TCDD). However, extensive studies revealed that AhR interacts with a wide range of structurally diverse molecules that originate from the diet, the environment, the microbiota or are endogenously produced by the host itself ^5,6^. Many of the microbiota-derived AhR ligands result from tryptophan catabolism including indole, indole-3-acetic acid, indole-3-aldehyde^7,8^. Upon ligand-binding, cytoplasmic AHR translocates in the nucleus, dimerizes with AhR nuclear translocator (ARNT) and initiates the transcription of target genes with promoters containing a xenobiotic-response element (XRE) sequence, such as cytochrome P450 family 1A1 (*CYP1A1*) and the repressor *AHRR*^9,10^.

AhR activation has been largely reported to be implicated in colonic stem cells proliferation, epithelial barrier functions and the regulation of a wide range of immune cell populations including intraepithelial RORγt^+^ innate lymphoid cell-3 (ILC3), γδT cells, T helper (Th)17/22 cells, intraepithelial, FOXP3^+^ regulatory T cells and antigen presenting cells^2–4,11,12^. The AhR-dependent *IL22* expression by ILC3 regulates the release of antimicrobial peptides such as RegIIIγ and the expression of tight junctions molecules in IECs thus reinforcing the barrier and defence functions^13,14^. Interestingly, AhR signalling and known AhR ligands are low in inflammatory bowel diseases (IBD) patients, highlighting the clinical relevance of the AhR pathway in these pathologies^15,16^. AhR activation by ligand administration and AhR knock-down in mouse suppressed and enhanced DSS-induced colitis, respectively suggesting a beneficial effect of AhR activation in IBD^17,18^. An increasing number of bacterial metabolites have been shown to drive the AhR activation, with described protective effects against intestinal inflammation and pathogens colonisations, suggesting a possible role of this signalling pathway in the intestinal homeostasis^7^. This hypothesis has been further supported by a recent study showing that one IBD-associated single nucleotide polymorphism (SNP) within the *CARD9* gene, affects microbiota composition thus altering the production of bacterial AhR ligands and consequently intestinal inflammation^15^.

The relevant role of AhR in the maintenance of mucosal homeostasis has been largely focused on immune cells from the *lamina propria*, and the impact of AhR activation in intestinal epithelial cells (IEC) has only been starting to be unravelled. Emerging evidences highlight that AhR activation impacts also IECs responses contributing to intestinal homeostasis. AhR activation in IECs protects mice from colitis by enhancing IECs barrier functions via the increase of the IL10 receptor expression and the enhancement of tight junctions integrity through the regulation of *Notch1*^19,20^. Importantly, the excess of AhR ligands in the gut is detrimental to the host. In this context, a recent study in mice model nicely demonstrates that IECs play the role of gatekeeper *via* the expression of the AhR-regulated gene *Cyp1a1* encoding cytochrome P450 1A1 involved in AhR ligands clearance^21,22^. These studies highlighted the importance of AhR ligands in modulating host gut immune homeostasis and prompted us to identify new microbiota-derived activators of the AhR pathway in IECs. We thus tested the bacterial supernatants of over 100 bacterial species of the human microbiota on an AhR reporter system in human intestinal cell lines and found that butyrate-producing bacteria activate the AhR-dependent pathway. A recent work previously highlighted the ability of butyrate to enhance the expression of AhR-dependent genes through its histone deacetylase inhibitor (HDACi) properties^23^. We confirmed the butyrate-activating role at the transcriptional level on AhR-dependent genes in Caco-2 and HT-29 cell lines. Interestingly, other HDACi that could enhance AhR-dependent gene expression were unable to mimic the butyrate dependent-activation of the AhR reporter system, suggesting the existence of a second mechanism. We showed that (i) AhR ligand antagonists impaired the butyrate-induced activation of AhR reporter system, (ii) butyrate enhanced AhR nuclear translocation and (iii) *in silico* modelling of butyrate interaction with human AhR, highlighting for the first time that butyrate could act as a ligand of AhR.

## Results

### Metabolites derived from commensal bacteria enhanced AhR activity

**Figure 1:**
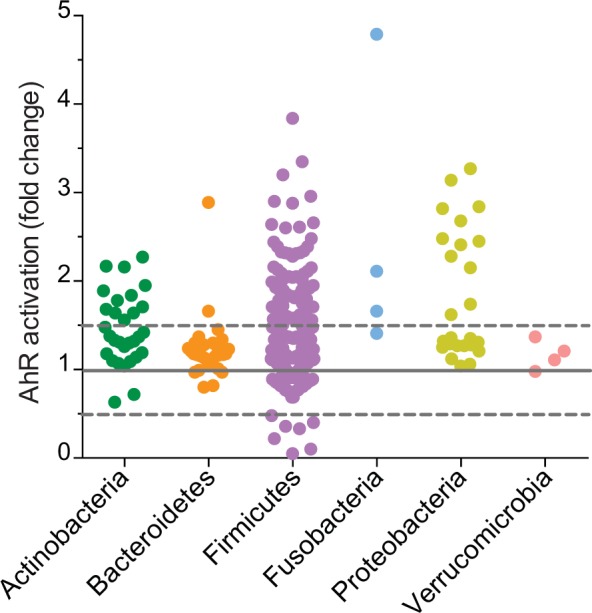
Screening of in-house strain collection of commensal bacteria on HT-29-AhR cell line. HT-29-AhR reporter cells were incubated with bacterial supernatants or relative non-inoculated bacterial media for 24 h (10% vol/vol). AhR activation was measured by luciferase activity and expressed as fold increase (±SD from triplicates) toward its control (non-inoculated bacterial media), sorted by Phyla.

In the gut, Aryl hydrocarbon receptor (AhR) ligands derive from diverse origins that include the intestinal microbiota as one of the main sources. To decipher which bacteria from human gut activate the AhR pathway, we performed a screening of commensal bacteria on a human intestinal epithelial cell line (HT-29-AhR) stably expressing an AhR-dependant reporter system. The AhR reporter system contains three copies of the DNA-binding domain motif recognized by AhR (xenobiotic-responsive element; XRE) driving the transcription of the luciferase reporter gene. Since AhR activators produced by bacteria are secreted in the intestinal lumen, we performed the screening of bacterial culture supernatants^8^. The screening included 132 bacterial strains, belonging to the major phyla of the human intestinal microbiota (Actinobacteria, Bacteroidetes, Firmicutes, Fusobacteria and Verrucomicrobia) (Supplementary Fig. S1, Supplementary Table S1) and grown under appropriate bacterial growth conditions (Supplementary Table S2). When possible, we cultured the bacteria in different media, to avoid biased results due to the rich composition of culture media, at least for almost all the bacteria activating the AhR reporter system. In our experimental set-up, AhR activation was detected in HT-29 cells challenged with some supernatants derived from Proteobacteria, Firmicutes, Fusobacteria and few Actinobacteria (Fig. 1).

### Butyrate activates AhR pathway in intestinal epithelial cell lines

**Figure 2:**
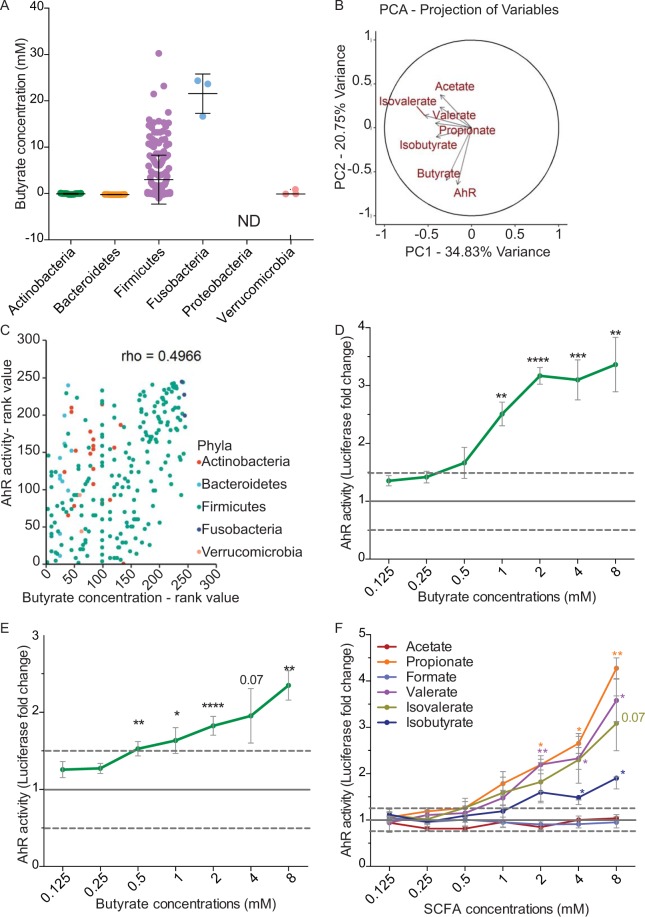
Butyrate activates AhR pathway. (**A**) HPLC quantification of butyrate produced by tested bacterial supernatants, sorted by Phyla (N.D.: Not Determined). (**B**) Principal Component Analysis (PCA) of quantified SCFAs and AhR activation for the entire data set. The axes PC1 and PC2 accounted for 34,83% and 20.75% of total data variation, respectively. (**C**) Spearman correlation analysis showing a positive relationship (rho = 0.4966) between AhR activity and butyrate concentration produced in commensal supernatants classified by rank values. HT29-AhR (**D**) or Caco2-AhR (**E**) reporter cells were incubated with a range of concentration of butyrate (0.125 mM to 8 mM). (**F**) HT-29-AhR reporter cells were incubated with SCFAs at concentrations rising from 0.125 mM to 8 mM. Data are expressed as luciferase fold (±SEM) of at least three independent experiments, normalised on untreated cells. ns: P > 0.05, *P ≤ 0.05, **P ≤ 0.01, ***P ≤ 0.001, ****P < 0.0001.

Despite the huge literature on AhR ligands, only few are commensal-derived molecules. Amongst them, indole and other tryptophan derivatives are produced by a wide range of bacteria including *Lactobacillus* (Firmicutes) and Proteobacteria^14,24^. In our screening we confirmed the AhR activation induced by some lactobacilli as well as by members of the Proteobacteria, probably due to the production of indole derivatives. Interestingly, among the bacteria not reported or not predicted to be indole producers, we identified some members of genera *Ruminoclostridium* and *Roseburia*, belonging to the Firmicutes phylum. These genera, together with other AhR-activating bacteria predicted to produce indole (*Clostridium* and *Lachnoclostridium*) share the ability to degrade diet-derived fibres, leading to the production of short-chain fatty acids (SCFAs)^25^. We thus hypothesized that SCFAs concentration in the supernatants of these bacteria could explain the activation of the AhR pathway as previously reported^23^. We therefore quantified the concentration of SCFAs in some bacterial supernatants (Supplementary Table S1 and Fig. 2A). A principal component analysis (PCA) of the complete dataset (SCFA concentrations and AhR activity) revealed that AhR activity and butyrate concentration were major contributors of total variability (Fig. 2B). Further Spearman correlation analysis showed a strong positive correlation between AhR activation and the production of butyrate quantified in commensal bacteria supernatants (rho = 0.4966, Fig. 2C).

To reinforce experimentally the observed correlations, we tested pure SCFAs at different physiological concentrations found in the intestine (ranging from 0.125 mM to 8 mM) on HT-29-AhR reporter cells (Fig. 2D,F)^26,27^. Among the tested SCFAs, we confirmed that butyrate was able to induce AhR activation in a dose-dependent manner, in HT-29-AhR cell line as well as in another intestinal reporter cell line, Caco2-AhR (Fig. 2D,E). Propionate, another abundant SCFA produced by bacteria, was also able to activate AhR in a dose-dependent manner in HT-29-AhR cells at a concentration starting from 1 mM (Fig. 2F). Interestingly, we also observed that valerate and the branched chain fatty acids iso-valerate, both described to influence epithelial physiology, were also able to activate AhR pathway in HT-29-AhR cell line at similar concentrations^28,29^ Iso-butyrate activated AhR only at 8 mM a concentration rarely reached in the intestine while acetate, the most abundant SCFA produced by commensal bacteria showed activation at high concentration (20 mM, Fig. 2F and Supplementary Fig. S2). Apart for acetate, the SCFA concentrations inducing AhR activity were consistent with the final SCFAs concentrations on bacterial supernatants measured in the screen thus reinforcing our hypothesis (Supplementary Table S1).

**Figure 3:**
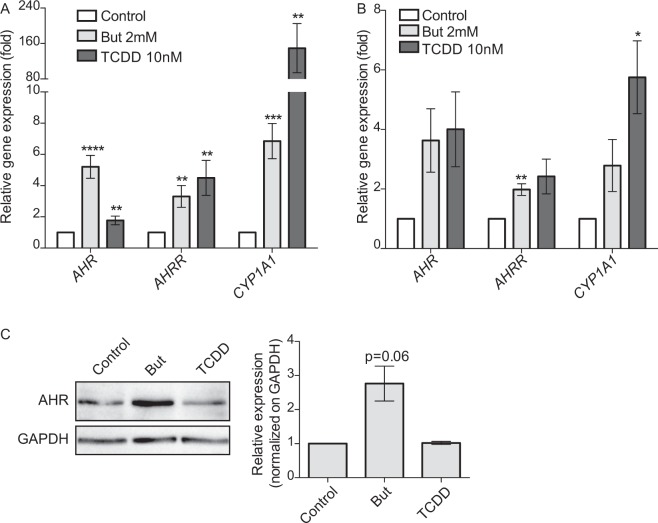
Butyrate activates the expression of AhR-regulated genes. The expression of AhR-regulated genes: *AHR*, *CYP1A1* and *AHRR* on HT-29 cells (**A**) and Caco-2 cells (**B**) treated with butyrate 2 mM or TCDD 10 nM during 6 h was determined by qRT-PCR. *AHR*, *CYP1A1* and *AHRR* relative expression to control is determined by the 2^−ΔΔCt^ method using *β-actin* for normalisation. (**C**) Up-regulation of AhR protein level by butyrate. HT-29 cells were incubated with butyrate 2 mM or TCDD 10 nM for 24 h. Total cytoplasmic extracts were blotted (Western Blot) for AhR (left panel) GAPDH was used as loading control. Relative quantifications from three independent experiments are expressed as fold-change to un-stimulated control of AhR protein normalised on GAPDH level (right panel). Full-length blots are presented in Supplementary Fig. S4. Data are expressed as means ± SEM of at least three distinct experiments, performed in triplicate. ns: P > 0.05, *P ≤ 0.05, **P ≤ 0.01, ***P ≤ 0.001, ****P < 0.0001.

Moreover, we showed by qRT-PCR that AhR-regulated genes, *CYP1A1*, *AHR* and *AHRR*, were up-regulated by butyrate both in HT-29 and Caco2 cells confirming that this SCFA activated AhR pathway (Fig. 3A,B). We confirmed the butyrate-driven up-regulation of AHR at the protein level by Western-Blot analysis in HT-29 (Fig. 3C).

### AhR activation by butyrate is independent of the SCFA receptors GPR41, GPR43, GPR109a and the SCFAs transporter MCT-1

**Figure 4:**
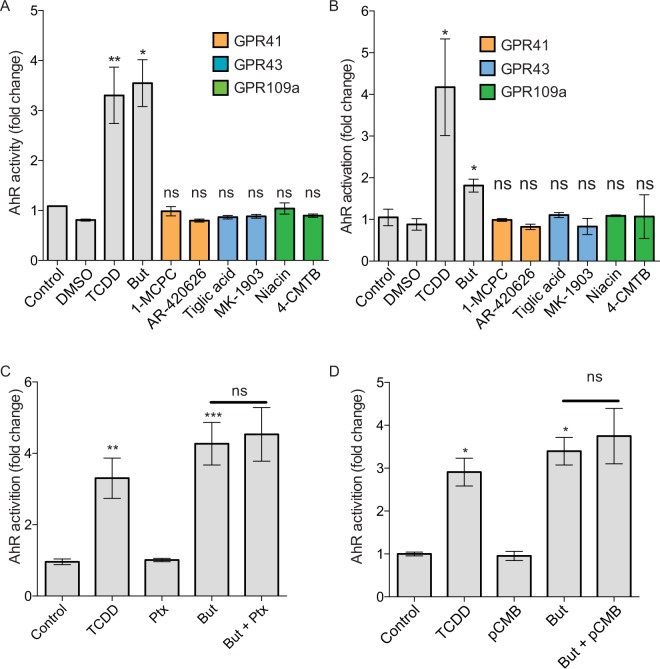
Mechanism involved in AhR activation by butyrate is independent of GPR and MCT-1. HT-29 (**A**) and Caco-2 (**B**) AhR reporter cells were stimulated for 24 h with GPR agonists. GPR41 (orange): 1-MCPC (1 mM), AR-420626 (1 µM); GPR43 (blue): Tiglic acid (1 mM), MK1903 (1 µM); GPR109a (green): Niacine (1 mM), 4-CMTB (1 µM). (**C**) HT-29-AhR reporter cells were incubated with Gα_i_-subunit inhibitor, Pertussis toxin (Ptx, 0.2 μg/mL) prior a 24 h butyrate stimulation (2 mM). Vehicle (Glycerol) was used as control. (**D**) HT29-AhR reporter cells were incubated during 24 h with a MCT-1 inhibitor, *p*-chloromercuribenzoic acid (pCMB, 10 uM), prior stimulation with butyrate (2 mM). AhR activation was measured by luciferase activity and expressed as fold increase means (±SEM) of at least three independent experiments, normalised on un-treated cells. ns: P > 0.05, *P ≤ 0.05, **P ≤ 0.01, ***P ≤ 0.001, ****P < 0.0001.

Butyrate, like other SCFAs, activates eukaryotic cells through two main mechanisms: activation of specific G-protein coupled receptors (GPCR: GPR41, GPR43, GPR109a) and inhibition of histone deacetylases (HDAC)^30–34^. Considering that the three G-protein coupled receptors are expressed in HT-29 and Caco-2 cells (Martin-Gallausiaux *et al. in press*), we firstly tested if the butyrate-induced AhR activation could be mediated by GPRs signalling by stimulating HT-29-AhR and Caco2-AhR cells with known GPRs agonists, targeting GPR41, GPR43 and GPR109a. For each GPR, two agonists were tested (GPR41: 4-CMTB and Tiglic acid; GPR43: AR420626 and 1-MCPC; GPR109a: Niacine and MK1903). Interestingly, none of the tested agonists induced AhR activation in luciferase reporter system suggesting that these GPRs are not involved in the butyrate-induced activation in HT-29-AhR and Caco-2-AhR cell lines (Fig. 4A,B). GPR41, GPR109a are both Gαi coupled receptors whereas GPR43 is a Gαi and Gαq coupled receptor. To further confirm our observation, we used an inhibitor of the Gαi pathway inhibitor: the pertussis toxin (Ptx) in HT-29-AhR (Fig. 4C). No impact on the butyrate-induced AhR activation was detected in cells when the Gα_i_ subunit was inhibited, further confirming that these GPRs were not involved in the observed AhR activation.

Butyrate uptake by the monocarboxylate transporter MCT-1 has been described as crucial for the GPR-independent regulation of a wide range of genes by butyrate^35^. To assess if MCT-1 participated in the activation of AhR by butyrate, a well-described inhibitor of MCT-1 transporter, pCMB, was tested on HT-29-AhR cells (Fig. 4D). The inhibition of MCT-1 transporter did not affect the ability of butyrate to activate AhR reporter system, suggesting that MCT-1 was not involved in this process.

### Activation of the AhR signalling pathway by butyrate is independent of its role as inhibitor of HDAC

**Figure 5:**
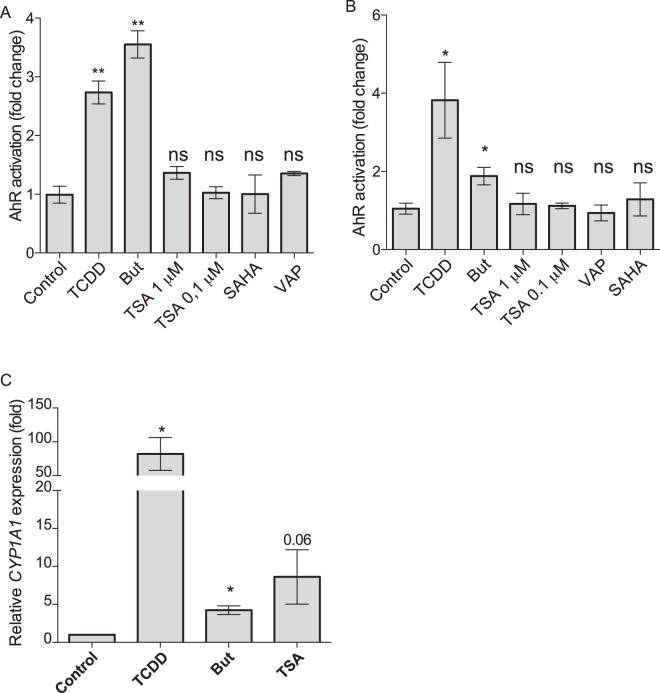
Impact of HDAC inhibitors on AhR reporter system and *CYP1A1*. HT29 (**A**) and Caco-2 (**B**) -AhR reporter cells were stimulated with HDAC inhibitors for 24 h. Trichostatin A (TSA, 0.1 μM and 1 μM), Vorinostat (SAHA 5 µM), Valproic acid (VAP 5 mM), butyrate (But, 2 mM) and TCDD (10 nM). (**C**) Relative expression to control of *CYP1A1* on HT-29 cells treated with butyrate 2 mM, TCDD 10 nM or TSA 0.1 μM during 6 h. *CYP1A1* expression induced by drugs is expressed as relative to control expression and is determined by the 2^−ΔΔCt^ method using *β-ACTIN* as control gene. AhR activation was measured by luciferase activity and expressed as fold increase means (±SEM) of at least three independent experiments, normalised on un-treated cells. ns: P > 0.05, *P ≤ 0.05, **P ≤ 0.01, ***P ≤ 0.001, ****P < 0.0001.

SCFAs, *via* their ability to inhibit lysine and histone deacetylases (HDAC), are potent modulators of histones and transcription factors acetylation that are well-documented regulatory mechanisms of gene transcription^30–32^. A recent study showed that SCFAs and other HDAC inhibitors (HDACi) enhanced the expression of *AHR*, *AHRR* and *CYP1A1 via* the increased level of histone acetylation of AhR-dependent genes^23^. These results prompted us to investigate the direct role of HDACi in the activation of AhR signalling pathway. To assess if butyrate impacted AhR pathway through its HDACi property, we tested three HDACi targeting a wide range of HDAC, trichostatin A (TSA), Vorinostat (SAHA) belonging to the hydroxamic acids family, structurally and metabolically unrelated to SCFAs and sodium valproate (VAP) belonging to the fatty acid family^36^. Interestingly, TSA, SAHA and VAP did not reproduce the butyrate-induced AhR activation in both HT-29-AhR and Caco2-AhR reporter system, suggesting that an additional mechanism leading to butyrate activation of the AhR pathway might exist (Fig. 5A,B). However, we could reproduce the activation of *CYP1A1* by TSA as observed by Jin *et al*. (Fig. 5C)^23^. Altogether, these results suggested that HDACi property of SCFAs was not involved in the butyrate-dependent activation of the AhR signalling pathway as monitored with reporter systems although HDAC inhibition might participate in the regulation of AhR-induced genes.

### Butyrate acts as an AhR ligand

**Figure 6:**
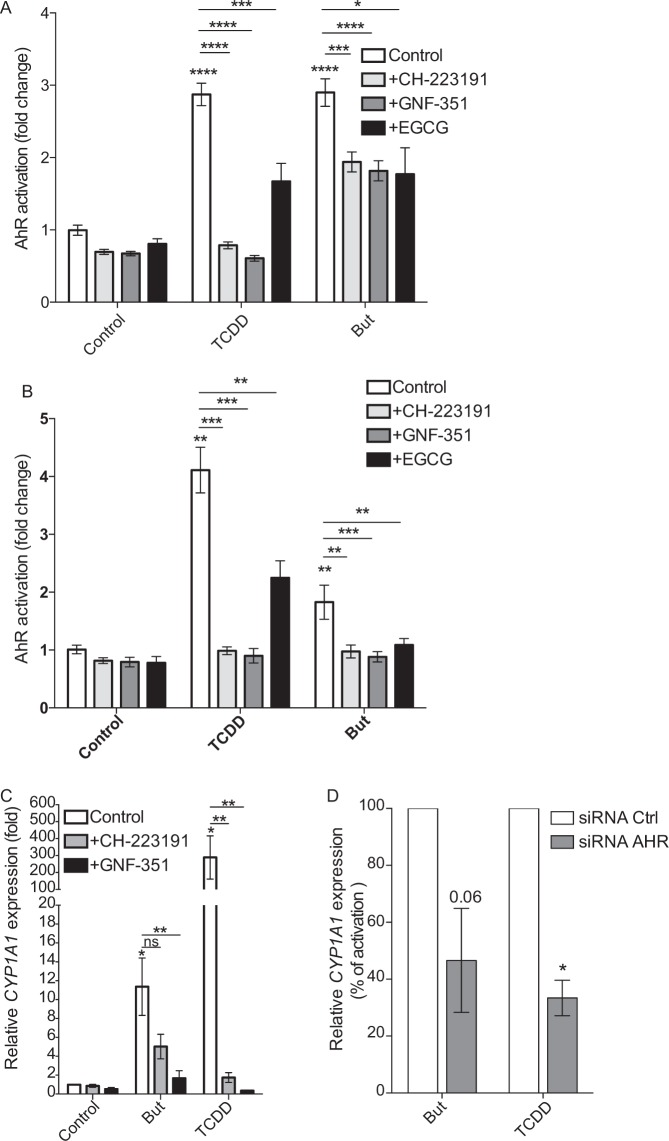
AhR antagonists inhibit AhR activation by butyrate. HT-29-AhR reporter cells (**A**) and Caco-2-AhR reporter cells (**B**) were incubated with AhR ligand antagonists (CH-223191 1 µM and GNF-351 1 µM) and an HSP90 inhibitor ((−)-Epigallocatechin-3-gallate, EGCG, 20 µM) prior stimulation with butyrate (But, 2 mM) and TCDD (10 nM) for 24 h. (**C**) Relative expression of *CYP1A1* on HT-29 cells treated during 6 h with 2 mM butyrate or TCDD 10 nM in presence or absence of AhR antagonists. *CYP1A1* expression is normalised to control expression (unstimulated cells) and is determined by the 2^−ΔΔCt^ method using *β-actin* as control gene. (**D**) Relative expression of *CYP1A1* on HT-29 cells transfected with control siRNA (white bars) or AhR siRNA (grey bars) and incubated during 6 h with 2 mM butyrate or TCDD 10 nM. *CYP1A1* expression is normalised to stimulated control expression (cells transfected with control siRNA and stimulated with either butyrate or TCDD), expressed in percentage and is determined by the 2^−ΔΔCt^ method using *β-actin* as control gene. Data are means ± SEM of at least three distinct experiments, performed in triplicate. ns: P > 0.05, *P ≤ 0.05, **P ≤ 0.01, ***P ≤ 0.001, ****P < 0.0001.

We showed that the two main mechanisms described for butyrate to activate transcription factors, HDACi and the activation pathway *via* GPRs, are not directly involved in the butyrate-induced activation of AhR signalling pathway monitored by reporter cell assay. Two groups described an original mechanism of gene regulation where butyrate acts as a ligand of the transcription factor PPARγ^37,38^. As AhR is a ligand binding transcription factors, we hypothesized that butyrate could act as an AhR ligand similarly to what was described for PPARγ. To demonstrate this hypothesis, we incubated butyrate-stimulated HT-29-AhR and Caco-2-AhR reporter cell-lines with two well-characterized antagonists of the AhR-ligand binding (CH-223191 and GNF-351) and one HSP90 inhibitor, described to avoid the downstream AhR-XRE binding, by blocking the release of HSP90 chaperon from the AhR complex (epigallocatechine-3-gallate, EGCG)^39–42^. The three tested inhibitors significantly decreased the butyrate-induced activation of AhR signalling pathway in both HT-29 and Caco-2 reporter cell lines (Fig. 6A,B). In addition, we confirmed in HT-29 cells that CH-223191 and GNF-351 were able to block *CYP1A1* expression induced by butyrate (Fig. 6C). The pivotal role of AhR in *CYP1A1* upregulation by butyrate was assayed using siRNA. As shown in Fig. 6D, we observed a diminished *CYP1A1* activation by butyrate and TCDD in cells treated with AhR siRNA compared to control siRNA. These results suggested that the activation of AhR signalling pathway by butyrate was due to a ligand-dependent AhR-XRE interaction, indicating a possible role of butyrate as a direct AhR ligand.

**Figure 7:**
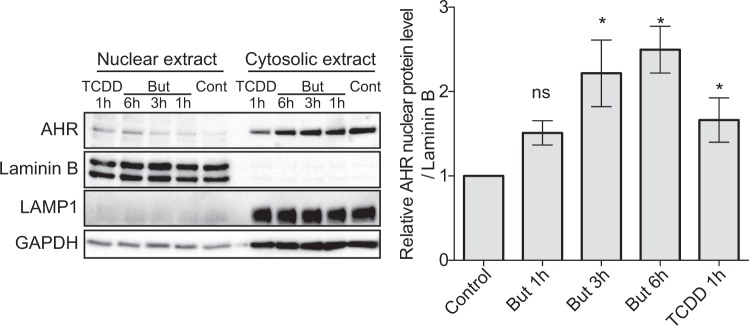
Butyrate stimulates AhR protein translocation in the nucleus. HT-29 cells were incubated during 1 h, 3 h or 6 h in presence of 2 mM butyrate or 1 h with TCDD. Nuclear and cytoplasmic extracts were blotted (Western Blot) for AhR protein expression. GAPDH and Lamp were used as control for cytoplasmic extract preparation; Lamin A/C as control for nuclear extract preparation (left panel). Relative quantification of AhR protein level in nuclear extracts from least six independent experiments is expressed as fold-change  (±SEM) to un-stimulated cells (control) after normalisation on Lamin A/C level. Full-length blots are presented in Supplementary Fig. S5. ns: P > 0.05, *P ≤ 0.05, **P ≤ 0.01, ***P ≤ 0.001, ****P < 0.0001.

It is well established that ligand binding triggers the accessibility of the nuclear localisation signal on the AhR N-terminus that consequently initiates the AhR nuclear translocation event^43^. Thus, additional evidence for the human AhR agonist potential of butyrate was obtained by performing nuclear translocation assay. Sub-cellular localisation of AhR in HT-29 cells incubated with butyrate (1, 3 and 6 h) and TCDD (1 h) was assessed by immunoblotting assay. We showed an accumulation of AhR proteins in the nucleus upon treatment with butyrate starting at 3 h and with a peak at 6 h, consistent with the action of an AhR agonist such as TCDD (Fig. 7).

Altogether, our results suggested that butyrate activation of the AhR reporter system relied on AhR translocation to the nucleus and consequent DNA-binding and that butyrate might act as a ligand of AhR.

### *In silico* modelling of butyrate interaction with AhR

**Figure 8:**
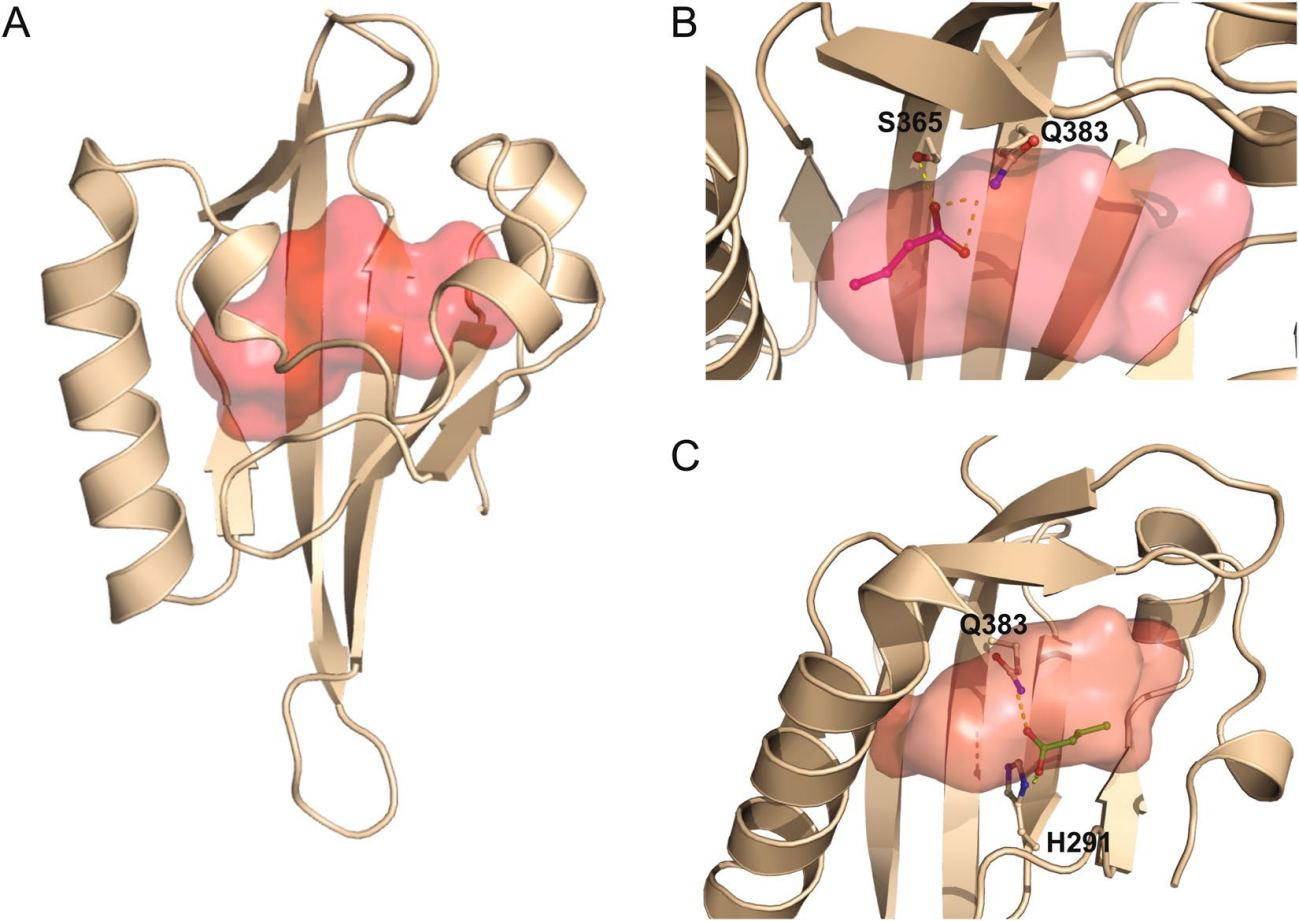
Modelling of butyrate binding to the ligand-binding pocket of human AhR. (**A**) Model of PAS-B domain of human AhR represented in cartoon coloured in wheat, the central cavity is represented as a red surface. Model of PAS-B domain of human AhR using 4ZP4 Crystal Structure of the Heterodimeric HIF-2a:ARNT complex obtain using RaptorX. The cavity has been defined by CARVER and HOLLOW. (**B**) Q383/S365 orientation. The residues Q383 and S365 are displayed as sticks and coloured by atom type with carbon in wheat. Butyrate is displayed as sticks and coloured by atom type with carbon in magenta. Hydrogen bonds are represented by white dashed lines. (**C**) Q383.H291 orientation. The residues Q383 and H291 are displayed as sticks and coloured by atom type with carbon in wheat. Butyrate is displayed as sticks and coloured by atom type with carbon in green. Hydrogen bonds are represented by white dashed lines. The figures were generated by PyMol.

Numerous AhR modulators, binding in the large central pocket of the AhR PAS-B domain, have been extensively studied using a combination of homology modelling and docking stimulations (for review see^44^). To support our results suggesting that butyrate acts as an AhR ligand, we performed a molecular docking simulation of the butyrate/AhR binding process. We first generated a homology model of human AhR ligand binding domain (AhR PAS-B) using HIF-2α PAS-B domain as template similarly to Bisson *et al*.^45,46^. The AhR PAS-B homology domain was obtained with RaptorX modelling software and the binding cavity was defined using HOLLOW and Carver softwares (Fig. 8A). Then, the model was minimized to avoid clashes between side chains. From the best scoring solutions cluster using HADDOCK, we observed different orientations of the butyrate inside the AhR PAS-B cavity. Based on four different docking experiments, two main orientations were found for butyrate in the pocket of human AhR PAS-B domain. Interactions between butyrate and AhR are dominated by polar contacts with the side chains of Q383 and S365, for the first orientation (Fig. 8B) and with side chains of Q383 and H291, for the second one (Fig. 8C). Interestingly, by comparing our results with published docking analyses of other ligands on human AhR PAS-B, the first proposed orientation for butyrate docking shared the same interacting side chains (Q383 and S365) with the docking of FICZ, proposed by Bisson *et al*.^45^. Overall these molecular docking analyses are coherent with our findings of butyrate as a direct modulator of AhR by ligand binding.

## Discussion

AhR is a ligand-activated transcription factor that is crucial for intestinal homeostasis by repressing inflammation and by maintaining the epithelial barrier in the gastrointestinal tract^3,47^. The gastrointestinal tract is a rich source of AhR ligands, which have been shown to induce AhR dependent responses and to protect the gut upon infection or induced colitis. Despite the huge literature on the structurally pleiotropic nature of AhR ligands, only few are commensal-derived molecules. Amongst them, indoles and other tryptophan derivatives are produced by a variety of bacteria including some Lactobacilli (Firmicutes) and Proteobacteria^14,24^. Interestingly, non-commensal bacteria have been reported to produce non-indole AhR ligands such as the phenazine derivative from *Mycobacteria* and the 1,4-dihydroxy-2-naphthoic acid from the probiotic *Propionibacterium freudenreichii*^13,48^. Given the abundance and extensive metabolic capacity of gut microbiome, it is thus likely that metabolites apart from tryptophan derivatives are present in high concentration to stimulate AhR pathway.

By screening commensal bacteria supernatants, we identified butyrate as a potent activator of AhR pathway and AhR-dependent genes. Butyrate is a short chain fatty acid (SCFA) that derived from bacterial fermentation of dietary fibers. Butyrate, *via* its role as inhibitor of lysine/histone deacetylase (K/HDACi), influences expression of a large variety of host genes in the colon, including some encoding for immune proteins^49^. Previous studies described butyrate as a regulator of AhR-dependent genes thought its role as histone deacetylase inhibitors (HDACi)^23,50^. However, these studies assessed the butyrate effect through induction of AhR transcriptional targets and not directly on the AhR receptor. Our results are in accordance with these studies as we showed that HDACi and butyrate upregulate *CYP1A1* expression. However, we demonstrated that butyrate does not impact AhR-dependent gene expression solely by its HDACi properties. Indeed by using an AhR reporter system in HT-29 and Caco2 cells, we showed that butyrate, and not HDAC inhibitors, activates AhR signalling pathway.

To decipher the mechanism of butyrate-dependent AhR activation, we investigated the implication of butyrate specific G-protein coupled receptors (GPR41, GPR109a and GPR43) and main transporter, monocarboxylate transporter 1 (MCT1)^51–53^. By using agonists and inhibitors, we showed that AhR activation by butyrate was not mediated by those GPRs or MCT1. Many studies evidenced that the predominant biological activities of the AHR are through ligand binding. Despite the AhR association with xenobiotic compounds, structurally diverse metabolites from the diet, bacteria or produced by the host have been reported as capable of binding to human AhR^6,54^. Our experimental results suggest that butyrate acts as an AhR ligand leading to the activation of the AhR pathway. Moreover, structural modelling of the binding of butyrate to human AhR PAS-B supports this hypothesis as we observed two main orientations of butyrate in the ligand-binding cavity of AhR. Interestingly, one of these orientations showed polar contact of butyrate with the same side chains of PAS-B reported for the binding of FICZ to human AhR supporting our hypothesis that butyrate is a ligand of AhR^45^. Confirmation of these results by direct amino acids point mutations in the ligand-binding cavity of human AHR similarly to previous publication on mice Ahr would have support our hypothesis^55^. This is an original mechanism of butyrate-dependent modulation of host gene expression, which has only been reported for another ligand-dependent transcription factor, PPARγ. Indeed, two groups reported that PPARγ-dependent genes regulation by butyrate is independent of its HDACi properties and is mediated through a direct binding of the SFCA to this transcription factor^37,38^. The strong impact of butyrate on mucosal immune homeostasis has been largely documented in mice and *in vitro* models. In human, studies have shown lower concentration of SCFA and butyrate-producing bacteria (i.e. *Faecalibaterium* and *Clostridium*) in the gut of inflammatory bowel diseases (IBD) patients suggesting a relevant role of butyrate in intestinal health^56–58^. Butyrate and other SCFAs elicit most of their biological activities by binding to GPRs and by acting as HDAC inhibitors^49^. Our results demonstrate an additional mechanism where butyrate binds to AhR thus activating its signalling pathway and in synergy with its HDACi property leads to the expression of AhR-dependent genes. These results might be generalized to other SCFAs as most of them activated the AhR signalling pathway (Fig. 2 and Supplementary Fig. S2) and *in silico* modelling of propionate interaction with AhR showed an orientation in the pocket of human AhR PAS-B domain similar to butyrate involving H291 and Q383 (Supplementary Fig. S3). However additional experiments are needed to validate this point. Interestingly, SCFAs such as butyrate exhibit overlapping activities with AhR ligands on intestinal homeostasis and in IBD^59^. AhR ligands regulate epithelial IL-10 receptor α subunit (IL-10RA) expression that damped colitis by promoting epithelial wound healing^60^. Similarly, butyrate promotes epithelial barrier formation through IL-10RA induction on IECs^61^. Hence, it is possible that AhR mediates the butyrate-induced IL-10RA up-regulation. Considering the high quantities of SCFAs in the gut, it is likely that butyrate possibly in synergy with other bacterial-derived AhR ligands have a role in the physiological functions of AhR. Nevertheless, the validation of our results in human primary IECs or colonic organoids is a future challenge to further decipher the mechanisms of butyrate impact on AhR signalling pathway, as well as other ligand-dependent transcription factors.

In conclusion, we show that butyrate stimulates AhR-dependent genes through a direct AhR activation and probably in complement to its HDACi property in human intestinal cell. Our results suggest that butyrate acts as a ligand of AhR which is, to our knowledge, an original mechanism only been reported for another ligand-binding transcription factor PPARγ^38^.

## Materials and Methods

### Cell Culture of human colonic cell lines

The human epithelial cell lines HT-29 and Caco-2 were obtained from the American Type Culture Collection (ATCC, Rockville, MD). HT-29 were grown in RPMI 1640 GlutaMAX™ and Caco-2 in DMEM GlutaMAX™ medium supplemented with 10% and 20% of heat-inactivated fetal bovine serum (FBS, Lonza), respectively. Both media were supplemented with 50 U/mL penicillin, 50 U/mL streptomycin and 10%, 100 mM Hepes, 10 mM nonessential amino acids. HT-29 and Caco-2 were grown at 37 °C in a humidified 5% and 10% CO2 atmosphere, respectively. All culture media and supplements were supplied by Gibco (ThermoFisher). Mycoplasma contamination was regularly tested using MycoAlert (Lonza) and PlasmoTest (Invivogen).

### Production of Stable AhR-luciferase Reporter Cell-Lines

pGL4.43[luc2P/XRE/Hygro] (Promega) was used to establish HT-29-AhR and Caco-2-AhR reporter cell-lines by electroporation using the Nucleofector® device (Lonza) according to the manufacturer’s recommendations. Stable AhR reporter cell lines were selected using Hygromycin (600 μg/mL for HT-29 and 200 µg/mL for the Caco-2 cell line, InvivoGen) and validated using TCDD at 10 nM final concentration.

### Culture of commensal bacteria, preparation of supernatants and SCFA concentration assessment

132 human intestinal commensal bacterial strains (106 different species) from the in-house INRA-Micalis collection or from DSMZ were grown. Anaerobic culture conditions were done accordingly to the Hungate method^62^. Screened strains, corresponding growth media, optical densities (OD600), SCFA concentrations are listed in Appendix Table S1 and composition of home-made growth media is listed in Supplementary Table S2. Bacterial cultures were cultured to reach the maximum OD. Bacterial supernatants were harvest after centrifugation at 5,000 × g for 10 min and filtered on a 0.22 μm PES filters and stored at −80 °C. Quality controls were performed using Gram staining method, aerobic growth test and fresh observation on microscope. Non-inoculated bacteria culture medium served as control. Concentrations of SCFAs produced by cultured bacteria were measured by HPLC and gas chromatography as described by^63,64^.

### Luciferase Reporter and Cell Viability Assays

For the bacterial screening, HT-29-AhR cells were seeded at 3 × 10^4^ cells per well in white 96-well plates (Corning). After 24 h from seeding, cells were stimulated during 24 hours with 10 μL of bacterial supernatant or non-inoculated media in a total culture-volume of 100 μL per well (10% vol/vol). The screening was performed in triplicates and for almost all the samples; experiments were performed at least with two biological replicates. Additionally, when possible, some strains were grown in different bacterial media. For testing the effect of reagents on AhR activity on HT-29 and Caco-2 cells, 24 h after seeding the culture media was replaced with a non-FBS-supplemented RPMI or DMEM. The cells were then stimulated with 10 µL of reagents diluted in non-FBS conditions in a total culture-volume of 100 μL per well (10% vol/vol). Follow-up experiments were performed in triplicates and repeated at least three times. Luciferase activity was quantified as relative luminescence units (RLU) using a microplate reader (infinite® 200 plate reader, TECAN) and the Neolite™ (PerkinElmer) Luciferase Assay System according to the manufacturer’s instructions. The AhR activation was normalised on non-inoculated bacterial media or untreated/vehicle-treated cells for bacterial supernatants and tested reagents, respectively. The results were expressed as luciferase fold change. Cell viability was assessed by MTS measurement using the CellTiter 96 Aqueous One solution (Promega) according to the manufacturer’s recommendations.

### Reagents

All agonists, antagonists and drugs tested were dissolved in a proper vehicle (DMSO, glycerol, water, PBS or ethanol) following the manufacturer’s recommendations. The final concentration used for vehicles had not detectable effect on metabolic activity of the cells. Sodium salts of tested SCFAs were from Sigma and used in a range of concentration from 0.125 to 8 mM (20 mM for acetate). AhR agonist: 2,3,7,8-Tetrachlorodibenzodioxin (TCDD 10 nM, Sigma). GPRs agonists: GPR41: 4-chloro-α-(1-methylethyl)-N-2-thiazolylbenzeneacetamide (4-CMTB 1 µM, Tocris) and Tiglic acid (1 mM, Sigma); GPR43: N-(2,5-Dichlorophenyl)-4-(furan-2-yl)-2-methyl-5-oxo-1,4,5,6,7,8-hexahydro-quinoline-3-carboxamide (AR420626 1 µM, Cayman) and 1-methylcyclopropane carboxylate (MCPC 1 mM, Sigma); GPR109a: Niacine (1 mM, Sigma) and (4aR, 5aR)-4,4a,5,5a-Tetrahydro-1H-cyclopropa[4,5]cyclopenta[1,2]pyrazole-3-carboxylic acid (MK1903 1 µM, Tocris). Pertussis toxin (Ptx at 0.2 µg/mL, Sigma) was used as Gα_i_-subunit inhibitor. MCT1 inhibitor used was p-Chloromercuribenzoate acid (pCMB 100 μM, Sigma). HDAC inhibitors: Trichostatin A (TSA 0.1 and 1 µM, Sigma), vorinostat (SAHA 5 µM, Sigma) and valproic acid (VPA 5 mM, Sigma). AhR antagonists: CH-22319 (1 µM, Millipore/Calbiochem), GNF-351 (1 µM, Millipore/Calbiochem), (−)Epigallocathechin gallate (20 µM, EGCG, Sigma).

### Real-Time PCR

Cell lines were seeded in 12-well culture plates at densities of 0.5 × 10^6^ cells per well. The cells were seeded in FBS-supplemented media then, after 24 h, the media was replaced with a non-FBS-supplemented and cells incubated during 24 h before stimulation. After stimulation time of 6 h, total RNA was extracted using RNeasy mini-Kit (Qiagen) according to manufacturer’s recommendations. cDNA was synthesized from 2 μg of RNA using the High-Capacity cDNA Archive Kit (Applied Biosystems). qPCRs were carried out using a StepOne (Applied Biosystems) thermal cycler in a reaction volume of 20 μL with Taqman gene expression assay probes: *AHR*: Hs00169233_m1; *AHRR*: Hs01005075_m1; *CYP1A1*: Hs01054796_g1; *β-ACTIN*: Hs99999903_m1. *AHR*, *CYP1A1* and *AHRR* expression relative to control expression was determined by the 2^−ΔΔCt^ method using *β-actin* as control gene. Data are means ± SEM of at least three distinct experiments, performed in triplicate.

### siRNA assays

0.5 x 10^6^ HT-29 cells were seeded in 6-well culture plates on day 1 and 25 nM of siRNA were transfected with DharmaFect I on day 2 and 3, following the manufacturer’s instructions (Dharmacon). After 48 h of culture, 0.5 x 10^6^ HT-29 cells were seeded in 6-well culture plates (day 4). Incubation with butyrate (2 mM) and TCDD (10 mM) was at day 5 for 6 h prior qRT-PCR assay. siRNA SMARTpool ON-TARGETplus *AhR* siRNA (L-004990-00-0005) and Non-targeting Pool (D-001810-10-05) were from Dharmacon.

### Cytoplasmic and nuclear protein extraction

HT-29 cells were seeded at densities of 0.5 × 10^6^ cells per well in 12-well-plates. 24 h after seeding the media was replaced with a non-FBS-supplemented RPMI and cells incubated during 24 h prior to stimulation. When nuclear extracts were not needed, cells were washed twice and lysed in buffer (1% NP40, 150 mM NaCl, 50 mM Tris-HCL pH8, 5 mM EDTA, 1x Complete Protease Inhibitor Cocktail (Roche)). Nucleus were eliminated by centrifugation for 10 minutes 4 °C at 17500 g. For compartments separation, nuclear and cytoplasmic extracts were prepared with NE-PER Nuclear and Cytoplasmic Extraction Reagent Kit (ThermoFisher) according to the manufacture instructions. CER I and NER buffers were supplemented with protease inhibitor cocktail (cOmplete™ ULTRA Tablets, Mini, *EASY*pack Protease Inhibitor Cocktail, Sigma) prior to use.

### Western Blot analysis

Protein extracts were run in 10% SDS-PAGE gels and transferred onto PVDF membranes by liquid transfer (Transfer buffer: 192 mM Glycine, 25 mM TrisBase, 20% methanol) at 200 mA during 90 minutes. Membranes were blocked overnight in TBST + 4% BSA (Sigma). Primary antibodies were incubated overnight at 4 °C: anti-AhR (1:500, mouse mAb, clone RTP1, ThermoFisher), anti-Lamin A/C (1:2000, mouse mAb, Cell Signaling), anti-GAPDH (1:4000, mouse mAb, Santa Cruz), anti-Lamp1 (1:2000, mouse mAb, H4A3 from the Developmental Studies Hybridoma Bank (DSHB), H4A3 was deposited to the DSHB by August, J.T./Hildreth J.E.K. (DSHB hybridoma product H4A3). Secondary mouse horseradish peroxidase-coupled antibody (DAKO) was successively incubated at room temperature for 2 h before detection with the Clarity Western ECL Substrate using the Chemidoc MP System (Bio-Rad). Quantifications were performed using the image Lab software (Bio-Rad). AhR nuclear protein levels were normalised to Lamin A/C protein levels. Lamp1 and GAPDH were used as purification controls for the cytoplasmic proteins.

### Modelling of butyrate binding to AhR

For modelling the structure of the complex between AhR and butyrate or propionate, HADDOCK software was used^65,66^. HADDOCK is a highly successful modelling approach that makes use of structural knowledge when available to drive the docking procedure. In this case the crystal structure of the heterodimeric HIF-2α:ARNT complex (PDB code 4ZP4)^67^ was used as template in order to dock butyrate to human AhR similarly to Bisson *et al*., using RaptorX^45,46,68,69^. The cavity of AhR PAS-B was defined by CARVER and HOLLOW^70,71^. The figures were generated with PyMOL Molecular Graphic System, version 1.8 Schrödinger, LLC.

### Statistical analysis

Presented results are representative of at least three independent experiments. The PCA analysis and Spearman correlation were performed using R and RStudio software. Graphics were produced using Prism GraphPad software. The data distribution was tested using D’Agostino-Person omnibus normality test. Normally distributed data was checked using two-sided t test, otherwise, non parametric Wilcoxon signed-rank or Mann-Whitney tests were performed according to the data set. In all tests, ns: P > 0.05, *P ≤ 0.05, **P ≤ 0.01, ***P ≤ 0.001, ****P < 0.0001.

## Supplementary information

Supplementary Figure S1-S5

Supplementary Table S1 and S2
