# Supplementary material for: Identification of the novel role of butyrate as AhR ligand in human intestinal epithelial cells
Source: Sci Rep. 2019 Jan 24;9:643. doi: 10.1038/s41598-018-37019-2 (PMC6345974; doi:10.1038/s41598-018-37019-2)
Supplement: Supplementary file 1 — Supplementary Figure S1-S5 [file 41598_2018_37019_MOESM1_ESM.pdf]

Identification of the novel role of butyrate as AhR ligand in human intestinal epithelial cells.

Ludovica Marinelli[1,2], Camille Martin-Gallausiaux[1,2], Jean-Marie Bourhis[3], Fabienne Béguet-Crespel[1], Hervé M. Blottière[1, 4] and Nicolas Lapaque[1*].

1. Micalis Institute, INRA, AgroParisTech, Université Paris-Saclay, 78350 Jouy-en-Josas, France

2. Sorbonne Universités, UPMC Univ Paris 06, IFD, 4 place Jussieu, 75252, Paris, cedex 05, France

3. University Grenoble Alpes, CNRS, CEA, IBS, F-38000, Grenoble, France

4. MetaGenoPolis, INRA, Université Paris-Saclay, 78350 Jouy en Josas, France

* Corresponding author:

Dr Nicolas Lapaque

INRA-MICALIS UMR1319, Bat 442, Domaine de Vilvert 78350, Jouy-en-Josas, France.

e-mail: nicolas.lapaque@inra.fr

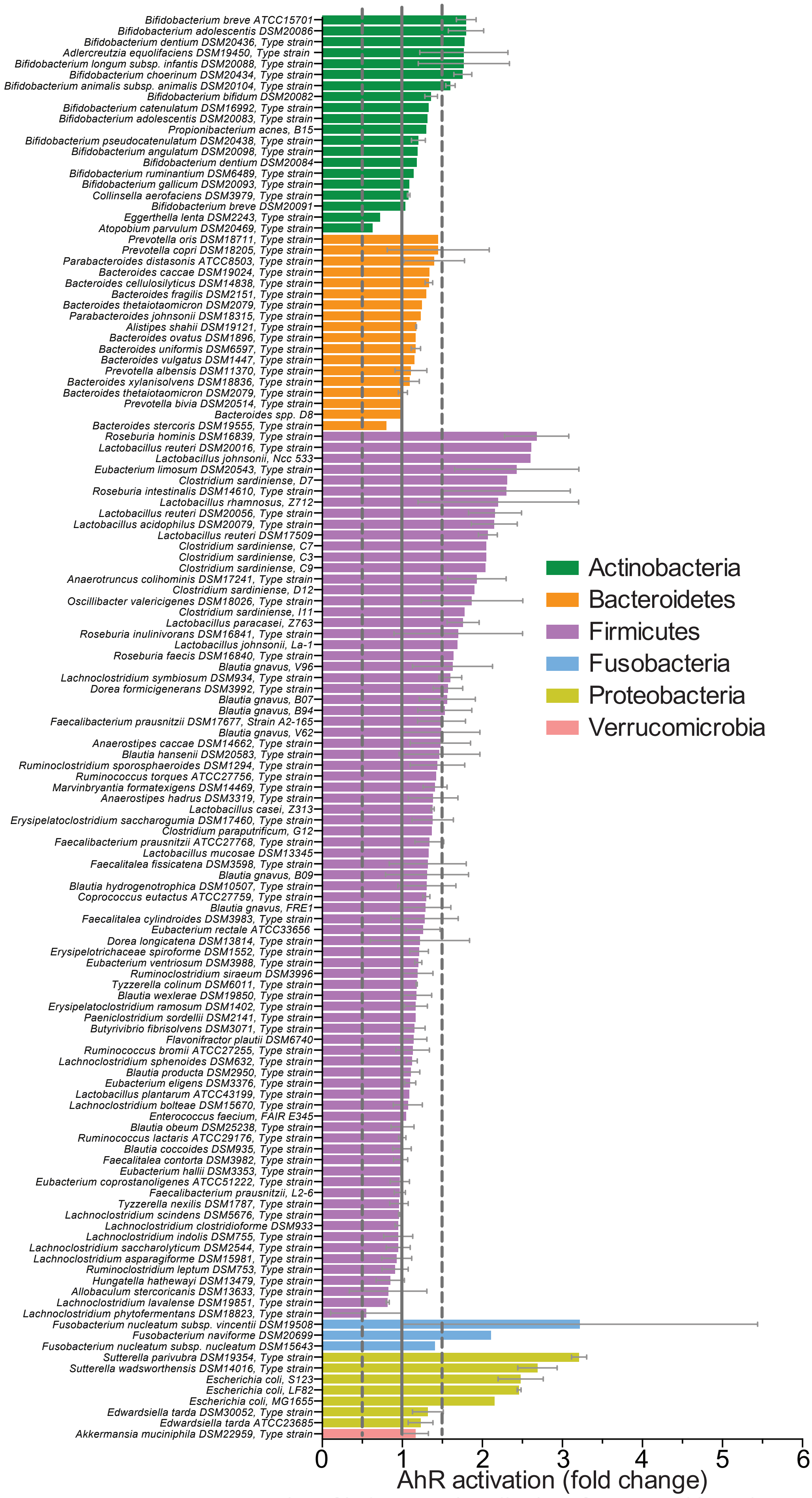

Supplementary Figure S1: Screening of in-house strain collection of commensal bacteria on HT-29-AhR cell line. AhR activation is expressed as the fold increase (± SD) toward its control (non-inoculated bacterial media), sorted by bacterial strain. Bacteria are sorted by response in decreasing order and grouped by Phyla (Actinobacteria in green, Bacteroidetes in orange, Firmicutes in purple, Fusobacteria in light blue, Proteobacteria in light green, Verrucomicrobia in pink). HT29-AhR cells were exposed to bacterial supernatants or relative non-inoculated bacterial media for 24h (10%vol/vol).

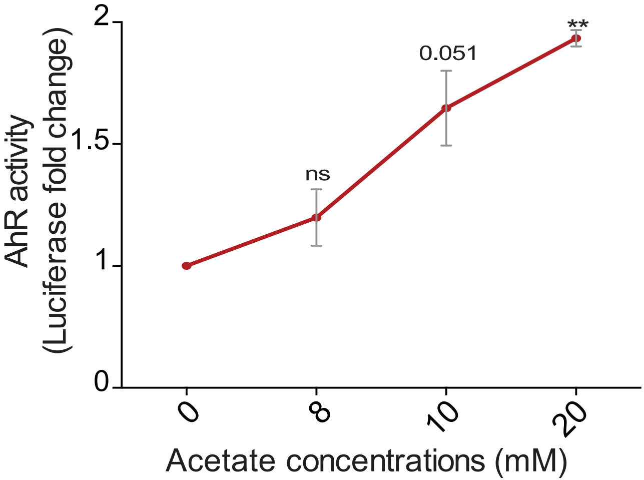

Supplementary Figure S2:

Effect of high concentrations of acetate on HT29-AhR reporter cell lines.

HT-29-AhR reporter cells were incubated with acetate at concentrations rising from 8mM to 20mM. AhR activation was measured by luciferase activity and expressed as fold increase means (± SEM) of at least three independent experiments, normalised on un-treated cells. ns: P>0.05, *: P≤0.05, **: P≤0.01, ***: P≤0.001, ****: P<0.0001.

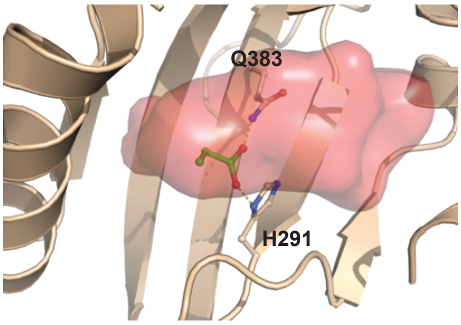

Supplementary Figure S3:

Modelling of propionate binding to the ligand-binding pocket of human AhR. Q383.H291 orientation is shown**.** The residues Q383 and H291 are displayed as sticks and coloured by atom type with carbon in wheat. Propionate is displayed as sticks and coloured by atom type with carbon in green. Hydrogen bonds are represented by white dashed lines. The figures were generated by PyMol.

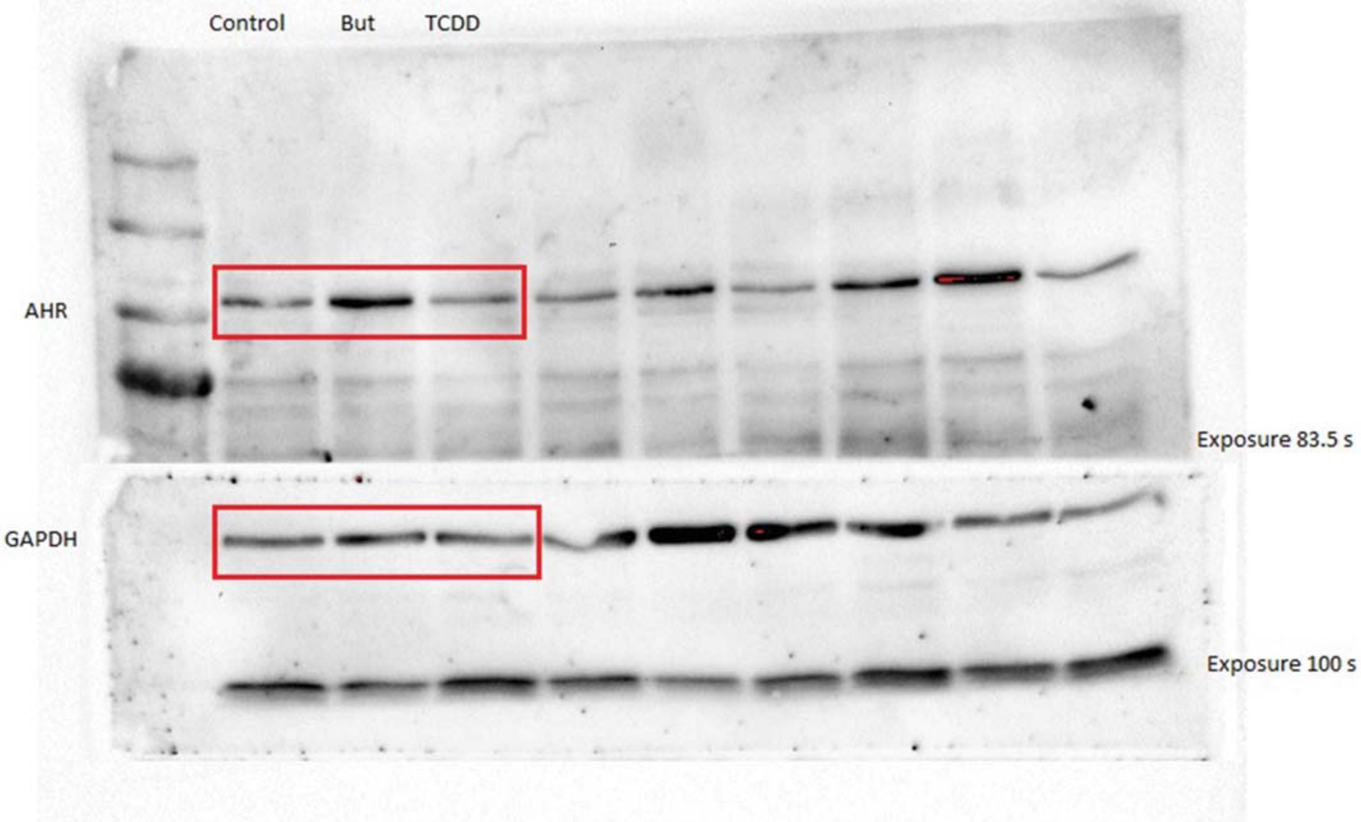

Supplementary Figure S4:

Full western blots of Figure 3C.

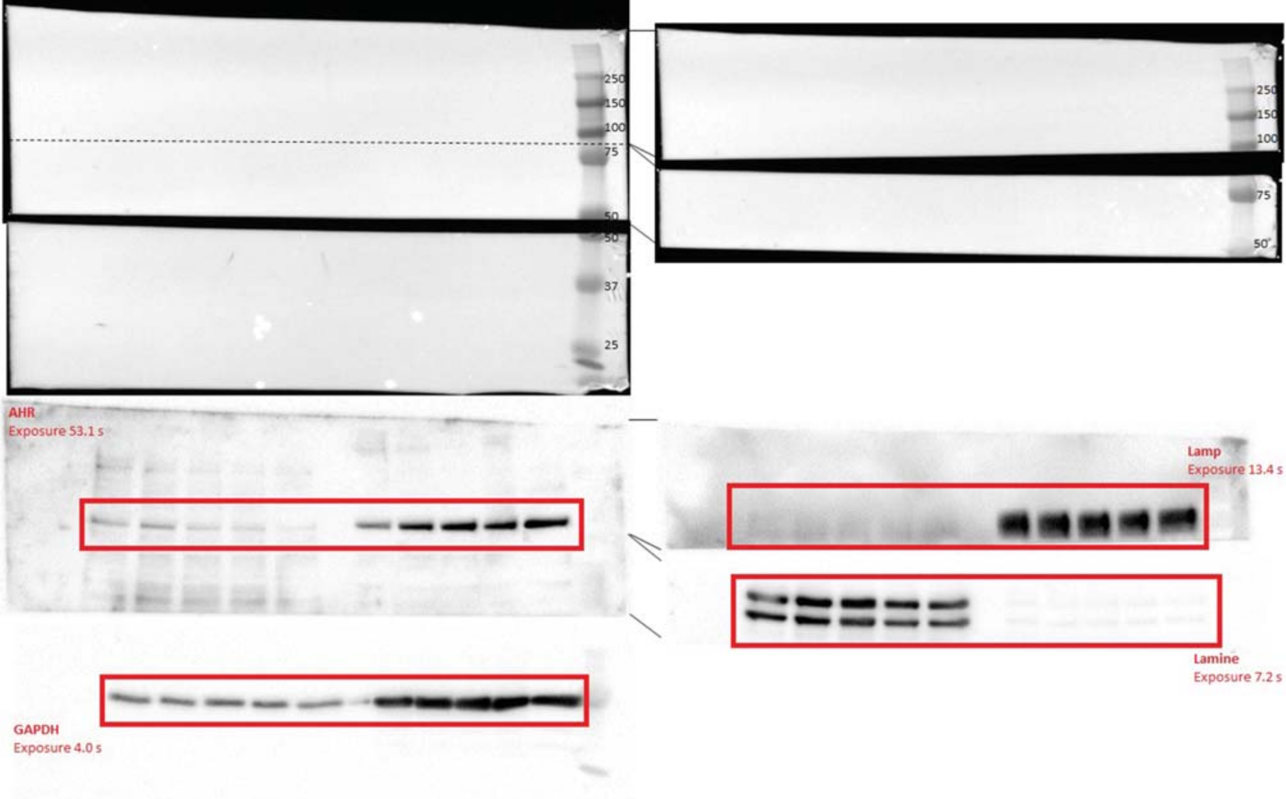

Supplementary Figure S5:

Full western blots of Figure 7.
